# Single-Port Laparoscopic Spleen Preserving Distal Pancreatectomy

**DOI:** 10.1155/2012/197429

**Published:** 2012-02-26

**Authors:** Stephen K. Y. Chang, Davide Lomanto, Maria Mayasari

**Affiliations:** ^1^Division of Hepatobiliary and Pancreatic Surgery, Department of Surgery, University Surgical Cluster, National University Hospital, Singapore 119228; ^2^Minimally Invasive Surgical Centre, Department of Surgery, National University Hospital, Singapore 119228

## Abstract

Single-port laparoscopic surgery has become increasingly popular, with widened indication to more types of surgery. This report will present our initial experience with spleen-preserving distal pancreatectomy technique through a small transumbilical incision using the single-port approach for a cystic tumor of pancreatic body. The surgery was done using specialized single-port instruments and normal laparoscopic instruments. The total operative time for this surgery is 233 minutes, and it was completed without drains. Patient was discharged from the hospital on the third day postoperatively in good condition.

## 1. Introduction

Distal pancreatectomy has been performed since early twentieth century [1].The first description of laparoscopic distal pancreatectomy was published by Soper et al. in 1994 [2] in animal model but since then many surgeons worldwide with better improvement of technologies, like ultrasonography, staplers, instrumentations, and so forth, have been applied safely in humans [3, 4]. In recent years, laparoscopic distal pancreatectomy had been performed for benign [5, 6], malignant [7], inflammatory lesion [8], and even for harvesting pancreatic donor for transplant [9]. Though technically feasible, this procedure is not frequently performed, probably due to the limited cases indicated for this procedure, the technical difficulty involved, and the high-tech devices required. Today indications for distal pancreatectomy include distal tumors (neuroendocrine and cystic lesions), chronic pancreatitis, and isolated pseudocysts.

In the past 10 years, minimal access surgery is increasingly popular and is moving towards further minimizing the surgical trauma by reducing numbers and size of the port. In the last few years, a novel technique called “Scar-less surgery” through a single-incision laparoscopic approach, has become one of the emerging technique. This technique is becoming popular especially for female patients due to the invaluable cosmetic results. In our institution, surgery using single port technique, such as appendicectomy, cholecystectomy, and hernia repair, is widely under investigation by randomized control trials. More complex operations with single-port technique are also being performed involving obesity surgeries, gastrectomies, liver resections, and so forth. Distal pancreatectomy may be another promising procedure that can be done through single-incision approach due to the wide range of instruments, energy sealing devices, and staplers available today.

This report will present our initial experience with spleen preserving distal pancreatectomy technique through a small transumbilical incision using the single-port approach.

## 2. Case Report

A 40-year-old female was found to have a 3.5 cm cyst at the body of the pancreas on ultrasound during a routine health screening. She had 2 previous laparoscopic procedures for pelvic inflammatory disease and excision of ovarian cyst.

**Figure 1:**
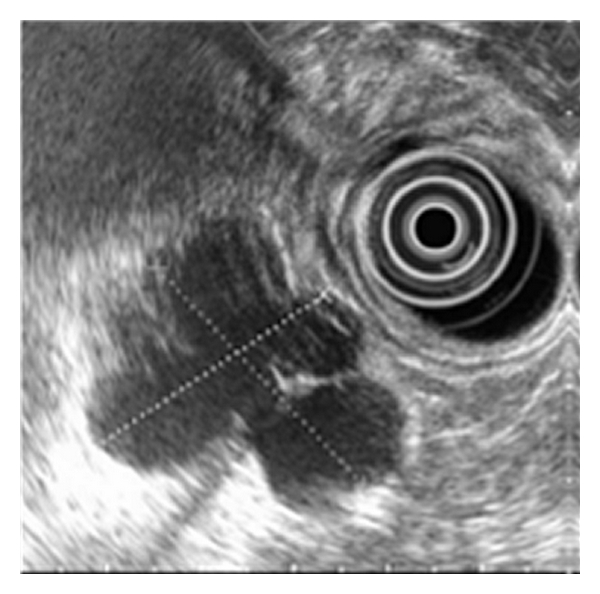
Endoscopic ultrasound image showing the cyst in pancreatic body.

A CT scan showed a complex cyst with septations measuring more than 3 cm and subsequent endoscopic ultrasound followed with fine-needle aspiration showed a multiloculated hypoechoic cystic lesion located at the body of pancreas with high Ca 19-9 of 148.2 U/mL (n.v. ≤ 37 U/mL), (Figure 1), suggestive of cystic mucin-producing neoplasm. She subsequently underwent spleen-preserving distal pancreatectomy via single-port approach.

## 3. Surgical Technique

Under general anesthesia, patient was placed in a French position with both arms tucked in. An SILS (Covidien USA) port was introduced through a 2 cm midline periumbilical incision, and three 5 mm ports were introduced into the SILS port.

Pneumoperitoneum was achieved, with pressure setting of 13 mmHg. A diagnostic laparoscopy was performed, using the 5 mm Endo-eye (Olympus, Japan) 30° telescope to confirm the absence of advance malignant disease. Out of the standard instrumentation, an Endograsp roticulator (Covidien AutoSuture, USA) was utilized during the surgery to avoid clashes and conflict between instruments and telescope and to improve triangulation.

**Figure 2:**
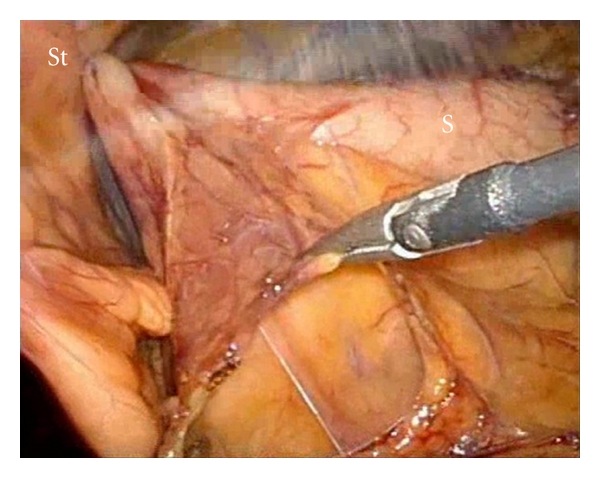
Opening of bursa omentalis. The stomach was retracted upwards with the help of stay sutures using prolene straight needle to the anterior abdominal wall. (St = Stay Sutures, S = Stomach.)

**Figure 3:**
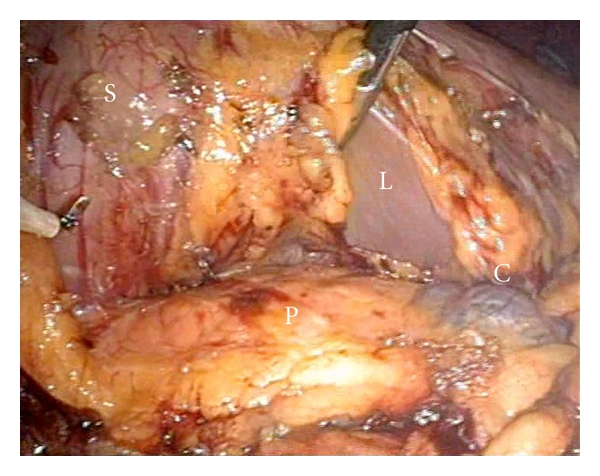
Exposure of pancreas. The lesion is seen at the right side of the picture. (C = cyst, P = pancreas, L = liver.)

The lesser sac was entered by opening the omentum along the greater curvature of the stomach using Ligasure (Covidien, USA), this allows the exposure of the pancreas as in standard technique. A total of three prolene straight needles stay sutures were placed superficially to the posterior gastric wall and slinged to the anterior abdominal wall to expose the pancreas (Figure 2). The cystic lesion was identified at the body of pancreas, measuring approximately 3 cm (Figure 3). Intraoperative laparoscopic ultrasound was used to confirm the lesion and that no other lesion was present.

**Figure 4:**
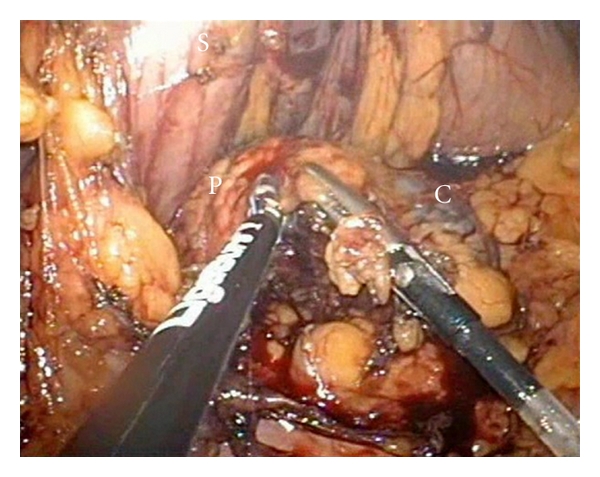
Transection of pancreatic neck using ligasure and roticulator endograsper.

**Figure 5:**
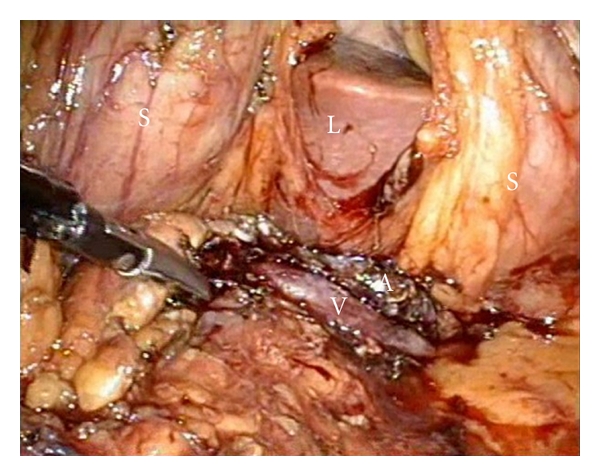
Tumor bed after resection. The splenic vessels (A = splenic artery, V= splenic veins) are seen intact in the horizontal manner.

After the lesion has been identified and assessed to be operable, the inferior edge of the pancreatic capsule is incised. Subsequently, a tunnel was created beneath the pancreatic neck from caudal to cephalad direction and freeing the pancreatic parenchyma from the splenic vessels. A cotton sling was passed through to lift the pancreas, and the pancreatic neck was then transected with the use of Ligasure (Figure 4) preserving the splenic vessels. A careful dissection of distal pancreas from medial to lateral approach was carried out with preservation of the main splenic artery and veins (Figure 5).

**Figure 6:**
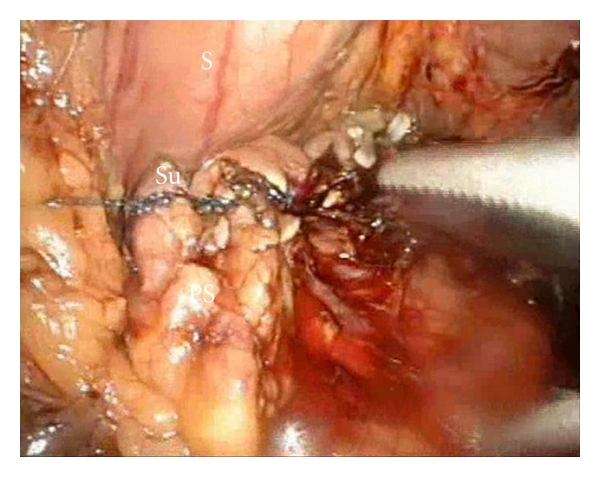
Pancreatic stump postsuturing (Su = sutures, P = pancreas).

**Figure 7:**
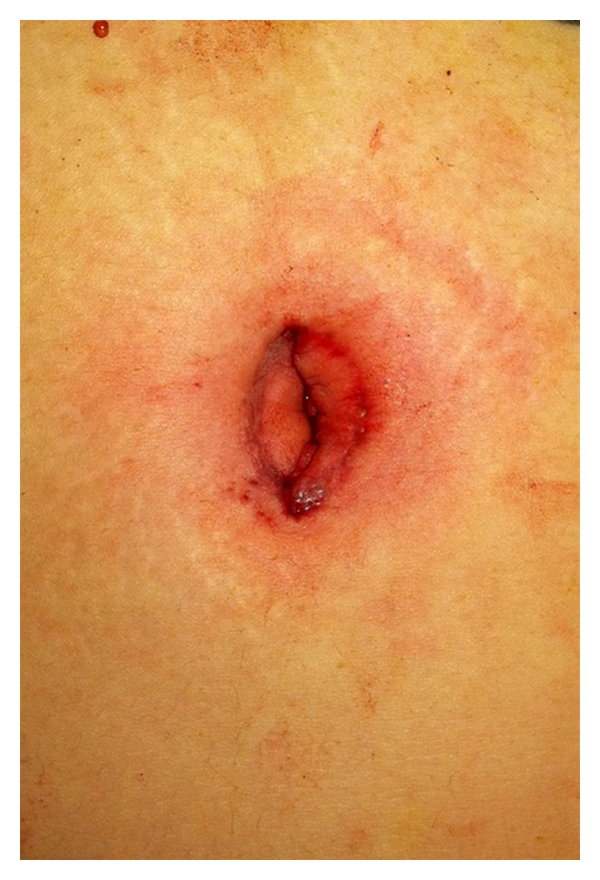
Postoperative wound.

Short transverse branches of the splenic artery and vein were individually isolated and sealed using Ligasure and the distal pancreatectomy was carried out by dissecting the specimen off its retroperitoneal attachments. The pancreatic stump was reinforced with continuous suture using V-lock suture-needle (Covidien, USA, Figure 6) involving the pancreatic duct. Afterwards, the prolene lifting sutures were removed and the specimen retrieved using bag retrieval (Applied Medical, USA) and delivered out through the umbilical wound (Figure 7).

The umbilical fascia was closed using 2.0 PDS sutures (Ethicon, USA), and no drains were inserted. Total operative time was 233 minutes, total blood loss was less than 100cc.

Patient recovery was uneventful. Liquid diet was started on first postoperative day before progressing to normal diet on the second postoperative day. Independent ambulation was achieved on the first postoperative day. She was discharged on the third postoperative day. Postoperative histopathology report was macrocystic serous cyst adenoma with free margin of the tumor.

## 4. Discussion

Distal pancreatectomy is not commonly done in many centers due to lack of suitable cases for this procedure. However, when indicated, laparoscopic approach is preferred than open. A meta-analysis [10] in 2010 showed that the minimally invasive approach has less morbidity and shorter hospital stay than open approach. Therefore, a laparoscopic approach should be considered as the first approach for distal pancreatectomy.

Single-port laparoscopic surgery [11–15] has been an emerging technique implemented and offered in simple cases such as appendicectomy and cholecystectomy worldwide in our institution. This approach may take longer to complete and require advance skills and dedicated instrumentations to compensate the lack of the triangulation as in conventional laparoscopy. In our experience, a combination of articulated grasper or dissector, sealing device like Ligasure, and telescope like Endoeye is necessary to overcome the clashes of instrumentations during single-port laparoscopic surgery. This allows a good dissection, traction, sealing and prevents instrument clashes within or outside of the abdomen. The options of using Ligasure advance, in this operation, was based on its ability to sealed vessels up to 6 mm and to have a thin tip for dissection. This is particularly important in keeping a bloodless view when dissecting the pancreas because of the rich blood supply of the organ and the tiny transverse branch of the splenic vessels. The operative time was 233 minutes, comparable to the average time used for conventional laparoscopic distal pancreatectomy of other series [10, 16].

The size of the lesion was 3 cm and is within the accepted indication for laparoscopic approach [16]. Probably for larger lesion (>3-4 cm), the single-port approach would not be appropriate, because of the need of a larger the incision to deliver the specimen out of the abdomen.

In our spleen-preserving technique, we carefully preserve both splenic vessels; this method is our preferred technique, since it avoids the splenectomy with all related intra- and postoperative complications as described by Warshaw [17, 18], like delivering a large organ out through the small port site, the risk of postoperative splenic infarction, and the postsplenectomy morbidity.

The postoperative recovery of the patient was uneventful and rapid with independent ambulation occurring on first day after surgery in keeping with the claimed advantages of minimal invasive over open approach.

## 5. Conclusion

Distal pancreatectomy is a complex procedure that was associated with high risk of complications and morbidity. The laparoscopic approach used has been well received with the experience of less complications and shorter hospital stay. The single-port laparoscopic distal pancreatectomy with spleen-preserving technique is a feasible and safe technique that can be done in selected cases and in highly qualified surgical centres.
